# Exosomes as Biomarkers and Therapeutic Agents in Neurodegenerative Diseases: Current Insights and Future Directions

**DOI:** 10.1007/s12035-025-04825-5

**Published:** 2025-03-17

**Authors:** Sam Dehghani, Ozgecan Ocakcı, Pars Tan Hatipoglu, Veli Cengiz Özalp, Atakan Tevlek

**Affiliations:** 1 Faculty of Medicine, Undergraduate Program, Atılım University, 06830 Ankara, Turkey; 2 Department of Medical Biology, Faculty of Medicine, AtıLıM University, 06830 Ankara, Turkey

**Keywords:** Exosomes, Neurodegenerative diseases, Biomarkers, Therapeutic agents, Blood–brain barrier, Alzheimer’s disease, Parkinson’s disease, ALS

## Abstract

Neurodegenerative diseases (NDs) like Alzheimer’s, Parkinson’s, and ALS rank among the most challenging global health issues, marked by substantial obstacles in early diagnosis and effective treatment. Current diagnostic techniques frequently demonstrate inadequate sensitivity and specificity, whilst conventional treatment strategies encounter challenges related to restricted bioavailability and insufficient blood–brain barrier (BBB) permeability. Recently, exosomes—nanoscale vesicles packed with proteins, RNAs, and lipids—have emerged as promising agents with the potential to reshape diagnostic and therapeutic approaches to these diseases. Unlike conventional drug carriers, they naturally traverse the BBB and can deliver bioactive molecules to affected neural cells. Their molecular cargo can influence cell signaling, reduce neuroinflammation, and potentially slow neurodegenerative progression. Moreover, exosomes serve as non-invasive biomarkers, enabling early and precise diagnosis while allowing real-time disease monitoring. Additionally, engineered exosomes, loaded with therapeutic molecules, enhance this capability by targeting diseased neurons and overcoming conventional treatment barriers. By offering enhanced specificity, reduced immunogenicity, and an ability to bypass physiological limitations, exosome-based strategies present a transformative advantage over existing diagnostic and therapeutic approaches. This review examines the multifaceted role of exosomes in NDDs, emphasizing their diagnostic capabilities, intrinsic therapeutic functions, and transformative potential as advanced treatment vehicles.

## Introduction

Neurodegenerative diseases (NDDs) are characterized by the progressive degeneration or death of neurons, which typically do not regenerate or undergo cell cycle completion [1]. As a result, neuronal loss in these disorders is irreversible, often leading to chronic and progressively worsening conditions. While the precise etiology of NDDs remains unclear [2], various risk factors have been implicated, including genetic predispositions, environmental influences, aging, and gene mutations [3]. Neuronal loss in NDDs is frequently associated with metabolic dysfunction or exposure to toxic agents [2], both of which trigger the activation of the innate immune system—a hallmark of many neurodegenerative conditions [4]. A key pathological feature common to most NDDs is the accumulation of misfolded protein aggregates, which serve as histological hallmarks of specific diseases and may overlap across multiple NDDs [5]. These aggregates, which form within neurons, are capable of propagating from affected cells to healthy neurons, contributing to the progression of neurodegeneration [5]. Prominent examples of neurodegenerative diseases include Alzheimer’s disease (AD), Parkinson’s disease (PD), Huntington’s disease (HD), frontotemporal lobar degeneration (FTLD), amyotrophic lateral sclerosis (ALS), spinocerebellar ataxias (SCA), and multiple sclerosis (MS), each distinguished by unique pathological features but often sharing common mechanisms of neuronal dysfunction and degeneration [6].

As the global population continues to age, with the proportion of individuals over the age of 65 being significant in 2019 and expected to nearly double by 2050, the prevalence of neurodegenerative diseases is projected to rise, emphasizing the need for research into their underlying mechanisms and potential therapeutic strategies [7]. Conventional diagnostic and therapeutic approaches to NDDs have considerable limitations. Biomarker-based diagnostics, such as cerebrospinal fluid (CSF) analysis and positron emission tomography (PET), sometimes involve invasive procedures and may be insensitive in early illness identification [8]. Pharmacological approaches, including as small-molecule medicines, enzyme inhibitors, and monoclonal antibody therapy (e.g., aducanumab), have been developed to target major pathological characteristics like amyloid-beta (Aβ) plaques in Alzheimer’s disease [9]. However, these techniques have limited selectivity, inferior effectiveness, and issues with BBB penetration, limiting their therapeutic potential. Furthermore, antibody-based therapies may elicit immunological responses, but their long-term efficacy is questionable due to varying patient responses and associated side effects [10].

In contrast, exosomes provide a revolutionary technique that solves several of these constraints. As naturally occurring extracellular vesicles, they provide a minimally invasive option for biomarker identification, with the possibility for real-time monitoring of disease development via liquid biopsies [11]. Furthermore, exosome-based treatments have a distinct advantage over synthetic drug carriers and viral vectors because they ensure targeted distribution of bioactive compounds with low immunogenicity. Exosomes generated from stem cells have shown neuroprotective capabilities, including regulating neuroinflammation, boosting synaptic plasticity, and improving Aβ clearance [12]. These processes go beyond the direct Aβ-targeting actions of monoclonal antibodies. Furthermore, exosome-mediated drug delivery enables the encapsulation of therapeutic molecules such as RNA-based medicines and neurotrophic factors, increasing bioavailability while limiting off-target effects [13]. These qualities establish exosomes as a paradigm-shifting tool in neurodegenerative disease research, overcoming current gaps in both early detection and treatment efficacy [14].

Exosomes are a subset of extracellular vesicles (EVs) that play a critical role in intercellular communication. Three main types of EVs exist: exosomes, apoptotic bodies (ApoBDs), and microvesicles (MVs), which differ in size and biogenesis [15]. Among these, exosomes are smallest, typically ranging from 30 to 150 nm in diameter, while ApoBDs vary between 50 and 5000 nm, and MVs range from 100 to 1000 nm [16]. The biogenesis of these vesicles follows distinct pathways. Unlike MVs, which bud directly from the plasma membrane, or ApoBDs, which are generated during programmed cell death, exosomes originate from endosomal compartments, forming intraluminal vesicles (ILVs) that are later released into the extracellular environment when multivesicular bodies (MVBs) fuse with the plasma membrane [17]. Additionally, exosomes are enriched with specific markers, including tetraspanins (CD9, CD63, CD81), ALIX, and TSG101, which distinguish them from MVs and ApoBDs that contain different surface markers and structural components [18].

Due to the essential function of intercellular communication in neurodegeneration, exosomes have become significant contributors to disease assessment and prospective treatment strategies [19]. Their ability to encapsulate and transport bioactive compounds, traverse the BBB, and influence cellular responses underscores their potential as biomarkers and therapeutic vectors in NDDs [20–23]. Additionally, recent studies indicate that exosome-like vesicles originated from plant sources possess structural similarities to mammalian exosomes and may facilitate neuroprotection by delivering bioactive compounds with antioxidant and anti-inflammatory properties [24, 25]. These plant-derived vesicles are reported as highly biocompatible, stable, and easily scalable, making them promising candidates for future therapeutic use in NDDs [26].

This review assesses the newly recognized function of exosomes in NDDs, emphasizing their potential as biomarkers and therapeutic agents, especially for early diagnosis, disease monitoring, and targeted medication administration. Their function in intercellular communication concerning neurodegeneration is examined, highlighting their distinctive capacity to cross the blood–brain barrier, rendering them particularly advantageous for therapeutic uses. Additionally, the constraints related to their clinical application are examined, and prospective research avenues that may advance the creation of exosome-based treatments for NDDs are emphasized.

## Overview of Major Neurodegenerative Diseases

AD represents the leading cause of dementia among elderly individuals, manifesting as progressive cognitive decline, memory impairment, and alterations in behavior [27]. Pathologically, AD is characterized by two defining features: extracellular amyloid-beta (Aβ) plaques and intracellular neurofibrillary tangles composed of hyperphosphorylated tau protein [28]. These aberrant protein aggregates interfere with synaptic communication, trigger neuroinflammatory responses, and ultimately result in extensive neuronal loss [29], particularly in the hippocampus and cerebral cortex—regions critically involved in memory and cognitive function [30]. The etiology of AD is multifactorial, encompassing genetic, environmental, and lifestyle influences [31]. Early-onset AD is strongly associated with mutations in the APP, PSEN1, and PSEN2 genes [32], whereas the APOE-ε4 allele is a major genetic risk factor for late-onset AD [33]. Other pathogenic contributors include oxidative stress, mitochondrial dysfunction, and chronic neuroinflammation [34]. Diagnosis of AD is based on a combination of clinical evaluation, neuroimaging, and the use of specific biomarkers, with a higher prevalence observed in women compared to men [35]. While current treatments, such as cholinesterase inhibitors and NMDA receptor antagonists, provide symptomatic relief, they do not halt disease progression [36]. Emerging therapies targeting amyloid-beta and tau proteins offer potential disease-modifying effects, yet their efficacy remains under investigation [37].

PD is a neurodegenerative disorder characterized by motor impairment due to the progressive loss of dopaminergic neurons in the substantia nigra [38]. Environmental factors, such as pesticide exposure, and genetic mutations in SNCA and LRRK2 are linked to disease development [39]. Dopamine depletion disrupts basal ganglia function, leading to the hallmark motor symptoms of PD [40]. The accumulation of Lewy bodies, primarily composed of misfolded α-synuclein, is the pathological hallmark [41]. Diagnosis is based on clinical motor assessments, supported by neuroimaging to exclude other conditions [42]. Treatment focuses on symptomatic relief with levodopa, dopamine agonists, and MAO-B inhibitors, while deep brain stimulation is used in advanced cases [43]. Key risk factors include age, family history, and exposure to neurotoxins, with an estimated ten million individuals affected globally, particularly those over 60 [39].

HD is a neurodegenerative disorder caused by a CAG repeat expansion in the HTT gene on chromosome 4, leading to the production of mutant huntingtin protein [44]. This aberrant protein aggregates in neurons, particularly within the cerebral cortex and basal ganglia, triggering progressive neurodegeneration and selective neuronal loss [45]. Clinically, HD presents with motor dysfunction and cognitive decline [46], and diagnosis is confirmed through genetic testing for the HTT mutation [47]. Neuroimaging techniques such as MRI often reveal cortical and basal ganglia atrophy [48]. Currently, there is no cure for HD [49], and management focuses on symptomatic relief, particularly for movement disorders and psychiatric symptoms, using agents such as tetrabenazine and antipsychotics [50]. Given its autosomal dominant inheritance pattern, individuals with an affected parent have a 50% risk of inheriting the mutation [51].

FTLD encompasses a group of neurodegenerative diseases characterized by progressive atrophy of the frontal and temporal lobes of the brain [52]. Symptoms vary based on the affected brain regions and may include personality changes, social withdrawal, speech difficulties, and impaired judgment [53]. Pathogenesis involves the accumulation of pathogenic proteins such as tau, TDP-43, and FUS, which lead to neuronal death and gliosis [54]. Familial cases are linked to mutations in genes like MAPT, GRN, and C9orf72, which promote abnormal protein aggregation [55]. Diagnosis is based on clinical assessment of behavioral and cognitive symptoms, supported by neuroimaging techniques like MRI and PET to observe brain atrophy patterns [56]. Genetic testing may be conducted in familial cases to identify specific mutations [57]. Currently, no cure exists, and treatment focuses on symptom management, with antidepressants and antipsychotics used to address mood and behavioral disturbances [58]. Supportive care, including speech and occupational therapy, plays a key role in enhancing quality of life for patients and their families. Risk factors for FTLD include advancing age and family history, with a higher incidence observed among individuals with affected first-degree relatives [59]. The prevalence is estimated at 15 to 22 cases per 100,000 people [60], primarily affecting individuals between 40 and 65 years of age [61]. Early diagnosis and intervention can significantly improve patient outcomes and care planning [62].

Amyotrophic lateral sclerosis (ALS) is a progressive neurodegenerative disease that primarily affects motor neurons in the brain and spinal cord, leading to muscle weakness, paralysis, and ultimately death [63]. Motor neurons control voluntary muscles involved in activities such as breathing, walking, and speaking [64]. As these neurons degenerate, muscle control deteriorates, while cognitive function is typically preserved, leaving patients fully aware of their condition [64]. Most ALS cases are sporadic with no clear genetic cause, while 5–10% are familial, often linked to mutations in genes such as SOD1, C9orf72, and TARDBP, which are associated with oxidative stress and protein misfolding [65]. Although no definitive environmental cause has been identified, factors like smoking, exposure to pollutants, and heavy metals may play a role in ALS development [66]. Patients often present with neuronal inclusions containing misfolded proteins such as TDP-43 [66]. Currently, there is no cure for ALS, and treatment focuses on symptom management [67]. Riluzole has shown efficacy in delaying disease progression by reducing glutamate toxicity, while edaravone may provide antioxidant benefits [68]. Multidisciplinary care, including respiratory support and physical therapy, is essential for improving quality of life [69]. Risk factors for ALS include male sex, advancing age (40–70 years), and family history, with occupational exposures [66] and military service also implicated [70]. ALS is considered a rare disease, affecting approximately 1–2 in every 100,000 people annually worldwide, with higher prevalence in North America and Europe [71]. Most patients survive 3–5 years post-diagnosis, although 10% live beyond 10 years [72].

SCA are a group of inherited neurodegenerative disorders characterized by progressive degeneration of the cerebellum, brainstem, and spinal cord, leading to ataxia—loss of coordination and balance [73]. SCAs are autosomal dominant, meaning a single mutated gene copy is sufficient to cause disease [74]. Over 40 subtypes have been identified, each linked to specific gene mutations, commonly involving CAG trinucleotide repeat expansions in genes such as SCA1, SCA2, SCA3 (Machado-Joseph disease), and SCA6 [75]. These mutations result in the formation of toxic protein aggregates, which cause neuronal death, particularly in the cerebellum [73]. Clinically, SCAs typically manifest in adulthood with symptoms including clumsiness, dysarthria, and ataxic gait, progressing to tremors, ocular movement difficulties, and fine motor impairments. Diagnosis is based on clinical assessment, MRI to detect cerebellar atrophy, and genetic testing to identify specific subtypes [76]. While no cure exists, management focuses on symptomatic relief, including physiotherapy to preserve mobility and medications for tremors and stiffness [73]. Risk factors include a family history of the disease, as SCAs are inherited [77]. The severity and onset can vary within families, often influenced by the length of the CAG repeat expansions. SCAs are rare, affecting 1–5 per 100,000 individuals globally, and the prognosis varies significantly depending on the subtype, with the progressive nature of the disease imposing a substantial burden on patients and healthcare systems [78].

MS is a chronic autoimmune disorder in which the immune system attacks the central nervous system, particularly affecting the brain, spinal cord, and optic nerves [79]. The immune-mediated destruction of the myelin sheath surrounding nerve fibers disrupts the transmission of nerve signals, leading to a range of neurological symptoms [80]. The damage results in scar tissue formation, or sclerosis, giving the disease its name. The cause of MS remains unclear, but it is thought to arise from a combination of genetic and environmental factors, including smoking, low vitamin D levels, and viral infections like Epstein–Barr virus [81]. MS is more common in regions farther from the equator and affects women two to three times more frequently than men [82]. Pathologically, MS is characterized by demyelination, axonal loss, and inflammation, resulting in lesions primarily in the spinal cord, optic nerves, and brain white matter [83]. Over time, neurodegeneration leads to irreversible disability [84]. MRI scans are essential for detecting areas of demyelination, and clinical evaluation and laboratory tests further assist in diagnosis [85]. MS management focuses on controlling symptoms, modifying disease progression, and enhancing quality of life [86]. Treatments include immunomodulatory drugs, such as interferon-beta, glatiramer acetate, and newer oral medications like fingolimod, as well as monoclonal antibodies like natalizumab and ocrelizumab. Progressive forms of MS remain challenging to treat, though ocrelizumab has shown promise in slowing disease progression [87]. Supportive care, including physiotherapy and medications for fatigue, pain, and spasticity, is crucial for symptom management [88]. Globally, MS affects over 2.8 million individuals, with an incidence of 100–150 cases per 100,000 people, typically presenting between the ages of 20 and 40 [89].

As seen across various neurodegenerative diseases, despite their distinct clinical manifestations, they share common pathological mechanisms, including protein aggregation, mitochondrial dysfunction, oxidative stress, neuroinflammation, and impaired cellular clearance pathways. These interrelated processes lead to neuronal loss and disease advancement, ultimately resulting in irreversible functional deterioration.

## Exosomes in Neurodegenerative Diseases

**Fig. 1:**
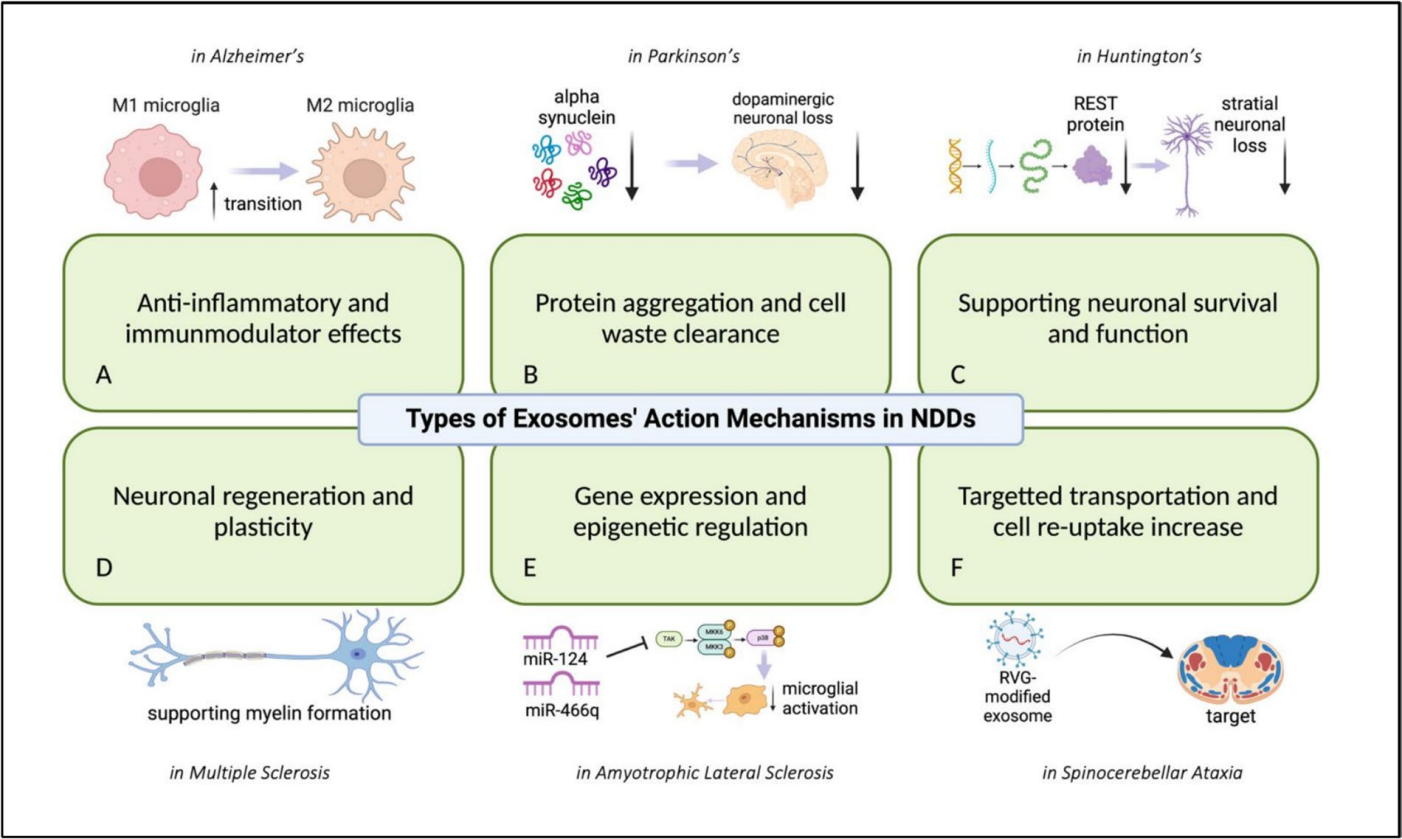
Types of exosomes’ action mechanisms in neurodegenerative diseases (NDDs). Exosomes contribute to **A** anti-inflammatory and immunomodulatory effects, **B** protein aggregation and cell waste clearance, **C** neuronal survival and function support, **D** neuronal regeneration and plasticity, **E** gene expression and epigenetic regulation, and **F** targeted transportation and enhanced cell re-uptake. Representative examples of these mechanisms are illustrated for Alzheimer’s disease, Parkinson’s disease, Huntington’s disease, multiple sclerosis, amyotrophic lateral sclerosis, and spinocerebellar ataxia

As research on NDDs progresses, increasing attention has been given to the role of exosomes in disease monitoring and treatment. Given their ability to transport biomolecules, traverse the blood–brain barrier, and indicate disease pathology, exosomes have become interesting candidates for biomarker identification and therapeutic applications in NDDs. Structurally, exosomes carry a diverse molecular cargo including proteins, lipids, RNA, and DNA, which they transport between cells, influencing a wide range of biological functions [90]. Due to their role in facilitating biomolecular transport, exosomes are essential in maintaining cellular homeostasis, as well as in regulating immune responses and inflammatory processes [91]. In recent years, exosomes have garnered significant interest in the field of neurodegenerative diseases (NDDs), such as Parkinson’s disease, Alzheimer’s disease, and Huntington’s disease, as both potential biomarkers and therapeutic agents [92]. Their ability to reflect the molecular changes occurring within diseased cells positions exosomes as promising non-invasive diagnostic tools [93]. By transporting disease-specific molecules such as proteins, RNAs, and lipids, exosomes can provide early insights into disease onset and progression, offering new avenues for monitoring neurodegenerative conditions [94]. Moreover, exosomes possess unique therapeutic potential, particularly in neurodegenerative diseases. Their natural ability to cross the blood–brain barrier makes them ideal candidates for delivering therapeutic agents, including drugs, RNA molecules, and gene-editing tools, directly to affected neurons [95]. This capability is crucial in developing targeted therapies aimed at modifying disease processes at the cellular level. Exosome-based treatments have the potential to modulate inflammation, reduce pathological protein accumulation, and restore cellular functions, thereby offering novel strategies for slowing or halting disease progression [96]. Exosomes exert their therapeutic effects in NDDs through multiple mechanisms, as illustrated in Fig. 1. In summary, exosomes represent a frontier in both the diagnosis and treatment of neurodegenerative diseases. Their distinctive ability to reflect cellular states and deliver targeted therapies underscores their potential in addressing the complex challenges posed by neurodegenerative conditions.

## Current Applications of Exosomes as Biomarkers in Neurodegenerative Diseases

Exosomes are small extracellular vesicles released by cells and are recognized as significant indicators in neurodegenerative disorders [97]. Exosomes, due to their capacity to encapsulate and transport specific proteins, lipids, RNAs, and DNAs, function as primary mediators of cellular communication and present substantial potential in the diagnosis of conditions such as Alzheimer’s and Parkinson’s [98]. Therefore, exosomes have been used as biomarkers in NDDs and recently several meta-analysis have been reported with regards to these properties [99–101]. In particular, exosomes found in the bloodstream represent an attractive biomarker for NDDs, owing to their relative abundance and accessibility in blood samples of the patients [102]. In Parkinson’s disease, blood exosomes contain α-synuclein, an essential biomarker for disease development [103]; in Alzheimer’s patients, increased amounts of amyloid-beta and phosphorylated tau proteins are present in circulating exosomes [104]. These blood-derived exosomal indicators enhance accuracy in diagnosis and enable noninvasive tracking of disease progression, resulting in early detection [105].

Although blood and cerebrospinal fluid (CSF) remain the primary sources of exosomes in current diagnostic methods, other biological fluids, including saliva, urine, sweat, breath, and tears, have attracted attention as non-invasive and promising alternatives. Saliva is a readily obtainable fluid that facilitates the collection of exosomes carrying amyloid-beta and tau proteins, which serve as unique biomarkers for Alzheimer’s, therefore contributing in early neurological diagnosis [106]. Urine is an effective medium for analyzing disease-specific peptides or proteins in urine exosomes, as it can potentially obtained non-invasively and in considerable amounts. Although urine is a distant site to the central nervous system, it provides an acceptable tool to evaluate systemic responses to neurodegenerative disorders [107].

**Fig. 2:**
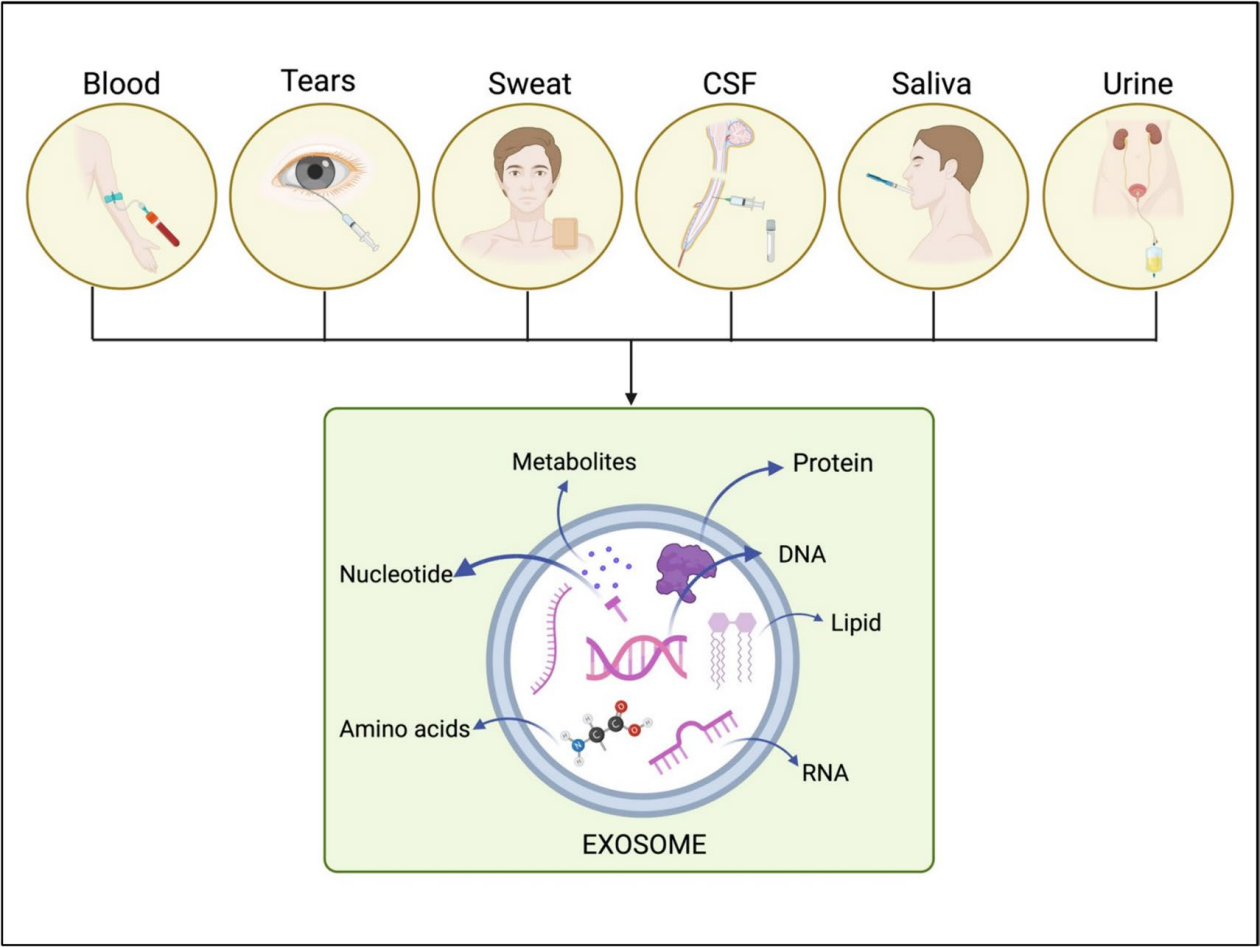
Depiction of exosome composition and its origins from bodily fluids. Exosomes transport many biomolecules, such as RNA, DNA, proteins, lipids, nucleotides, amino acids, and metabolites that enable intercellular communication. Common biological fluids, including blood, cerebrospinal fluid, saliva, perspiration, tears, and urine, provide accessible sources for exosome isolation and biomarker identification

While sweat enables continuous monitoring, particularly via wearable devices, it is a complicated sample type due to the low concentration of exosomes and the influence of ambient conditions, such as temperature, on exosome yields [108]. On the other hand, breath presents the opportunity for noninvasive, real-time biomarker analysis as a novel source of biological fluids; while primarily investigated for respiratory problems, research on breath exosomes may enhance neurodegeneration diagnosis with improved collecting methods [109]. Interestingly, tears are a highly interesting material for noninvasive biomarker studies, since tear exosomes from Alzheimer’s patients have demonstrated the presence of Alzheimer’s-specific biomarkers [110]. Each of these diverse fluid sources provides novel approaches to enhance exosome-based diagnostic techniques, ensuring minimal patient discomfort for the early diagnosis and monitoring of neurodegenerative disorders. Table 1 summarizes the most current sources, detection methods, and clinical applications of exosomal biomarkers in neurodegenerative diseases. On the other hand, Fig. 2 illustrates the many bodily fluids in which exosomes can be found as well as their enriched content.

**Table 1:** Exosomal biomarkers used in NDDs: source, detection methods, and clinical applications

Source	Types of NDDs	Exosomal biomarkers	Diagnostic tool	Clinical utility	Reference
Blood	Huntington’s disease	Full-length Huntingtin protein (HTT) (360 kDa), mutant Huntingtin fragment (~ 70 kDa)	Ultracentrifugation, western blotting method	Potential biomarker for diagnosis and monitoring of therapy efficacy	[111]
Blood	Frontotemporal lobar degeneration and GRN/C9orf72 mutation	Cathepsin D levels (ng/sEV)	ELISA, nanoparticle tracking analysis (NTA)	Diagnostic potential with high performance (AUC = 0.85); correlated with age of onset and progressive disease states	[112]
Blood	Alzheimer’s disease	Elevated levels of amyloid-β (1–42), phosphorylated Tau (p-Tau), synaptophysin, TNF-α, IL-1β, and GFAP	Chemical precipitation, transmission electron microscopy, nanoparticle tracking analysis, western blot, ELISA	Potential biomarkers for diagnosis, progression, and neuroinflammation	[113]
Blood	Amyotrophic lateral sclerosis	hsa-miR-34a-3p and hsa-miR-1306-3p for ALS patients with SOD1/C9orf72 mutations; hsa-miR-199a-3p and hsa-miR-30b-5p for patients with sALS	miRNA isolation, RT-qPCR	Potential biomarkers to distinct amyotrophic lateral sclerosis with mutant genes	[114]
Blood	Multiple sclerosis	CX3CR1⁺/UCHL1⁺, NMDAR2A⁺, and NFL⁺ microglial EVs	Nano flow cytometry	Potential biomarkers for diagnosing multiple sclerosis, distinguishing it from HTLV-1-associated myelopathy, and tracking disease duration	[115]
Blood	Dementia, Parkinson’s disease, multiple sclerosis	21 candidate protein biomarkers including APOE, CDH1, CDH13, CELSR2, CHI3L1, CHL1, CLEC3B, CLU, CST3, FGA, FGB, FGG, FLNA, H2AC20, MAN2A1, OLFM1, RAB7A, SLC2A3, TMEM198, TNC, and TREML1	Magnetic transferrin nanoparticles (MTNs) assay for specific exosome isolation, high-throughput proteomics	Promising diagnostic biomarkers for minimally invasive diagnosis of dementia, PD, and MS	[116]
Blood	Alzheimer’s disease	hsamiR-30b-5p, hsa-miR-22-3p, and hsa-miR-378a-3p	Western blot, RNA isolation, sequencing, qRT-PCR	Potential biomarkers for Alzheimer’s disease and candidate for developing miRNA panel	[117]
Blood	Frontotemporal dementia and Alzheimer disease	Ng, MFN-2, LAMP-2, golgin A4	Single molecule array (Simoa) technique, SDS-PAGE, immunoblotting	Identifying common mechanisms and differences among neurodegenerative diseases	[118]
Blood	Alzheimer’s disease	Aβ42, P‐tau, and T‐tau, GAP43, SNAP25	ELISA	identification of effective biomarkers for prediction of AD 5 to 7 years before cognitive impairment	[119]
Blood	Alzheimer’s disease	APOC3, APOH, C4BPα, CO3, KV230, AACT isoform 1, CO9, IGHM isoform 2, K2C6A	Precipitation-based isolation, column-based-isolation, mass spectrometry	Potential candidates for biomarker of Alzheimer disease and representation of disease progression	[120]
Cerebrospinal fluid, blood	Spinal muscular atrophy	Full-length SMN (flSMN) transcript	Droplet digital PCR, ultra-sensitive single molecule array	Potential biomarker and monitoring treatment response	[121]
Cerebrospinal fluid	Amyotrophic lateral sclerosis	Exosomal mRNAs with significant differential expression, including CUEDC2, TUBB3, and CAMK2A	Next-generation sequencing (NGS), DESeq2 for differential expression	Candidate biomarkers for ALS with potential in diagnosis and tracking disease mechanisms	[122]
Cerebrospinal fluid	Amyotrophic lateral sclerosis	increased levels of novel INHAT repressor (NIR); decreased levels of 11 unspecified proteins	Gel filtration chromatography, liquid chromatography-tandem mass spectrometry	Potential biomarker for ALS diagnosis and monitoring; indicates nucleolar stress involvement in ALS pathogenesis	[123]
Cerebrospinal fluid	Alzheimer’s disease, Parkinson’s disease, dementia, and their subtypes	37 disease-specific protein biomarkers identified	Label-free mass spectrometry	Potential biomarkers for differential diagnosis of dementia types and tracking of neurodegenerative progression	[124]
Cerebrospinal fluid	Huntington’s disease	Mutant Huntingtin protein (mHTT) levels in CSF as a pharmacodynamic biomarker	Antisense oligonucleotide therapy, viral expression techniques	Potential biomarker for monitoring mHTT reduction in the striatum; useful for evaluating therapeutic target engagement in the CNS for HD	[125]
Saliva	Alzheimer’s disease, cognitive impairment	Elevated levels of oligomeric amyloid-beta (Aβ), phosphorylated tau protein (p-tau), and CD63	Nanoparticle tracking analysis (NTA), automated western blot analyzer, fluorescent antibody quantification	Non-invasive, cost-effective screening method for AD and cognitive impairment progression	[126]
Saliva	Alzheimer’s disease	miRNA-485-3p associated with Aβ deposition	qRT-PCR, ROC curve analysis	Non-invasive biomarker for predicting Aβ-PET positivity; high diagnostic accuracy for Alzheimer’s disease	[106]
Urinary	Alzheimer’s disease	Aβ1–42 and P-S396-tau	Transmission electron microscopy; nanoparticle tracking analysis, ELISA	Potential biomarkers detection for early diagnosis of Alzheimer’s disease	[127]
Urinary	Alzheimer’s disease	miR-196b-5p, miR-339-3p, miR-34a-5p, miR-376b-3p, miR-677-5p, and miR-721	MicroRNA microarray, droplet digital polymerase chain reaction	Exploring differences in initial Aβ-plaque deposition and potential noninvasive biomarkers	[128]
Urinary	Alzheimer’s disease	Annexin 2 and Clusterin	BCA assay, liquid chromatography tandem mass spectrometry, nanoparticle tracking analysis, transmission electron microscopy, western blot, ELISA	Potential non-invasive source of biomarkers for potentially early intervention of Alzheimer’s disease	[129]
Tears and cerebrospinal fluid	Multiple sclerosis	TGFB1, ANGPT2, VEGFA proteins; beta-estradiol, progesterone hormones	Flow cytometry, FASP digestion, dynamic laser light scattering, liquid chromatography tandem mass spectrometry	Unveiling of cross talk between CSF and tears; new diagnostic perspectives for multiple sclerosis	[130]

## Therapeutic Mechanisms of Exosomes in Neurodegenerative Diseases

**Fig. 3:**
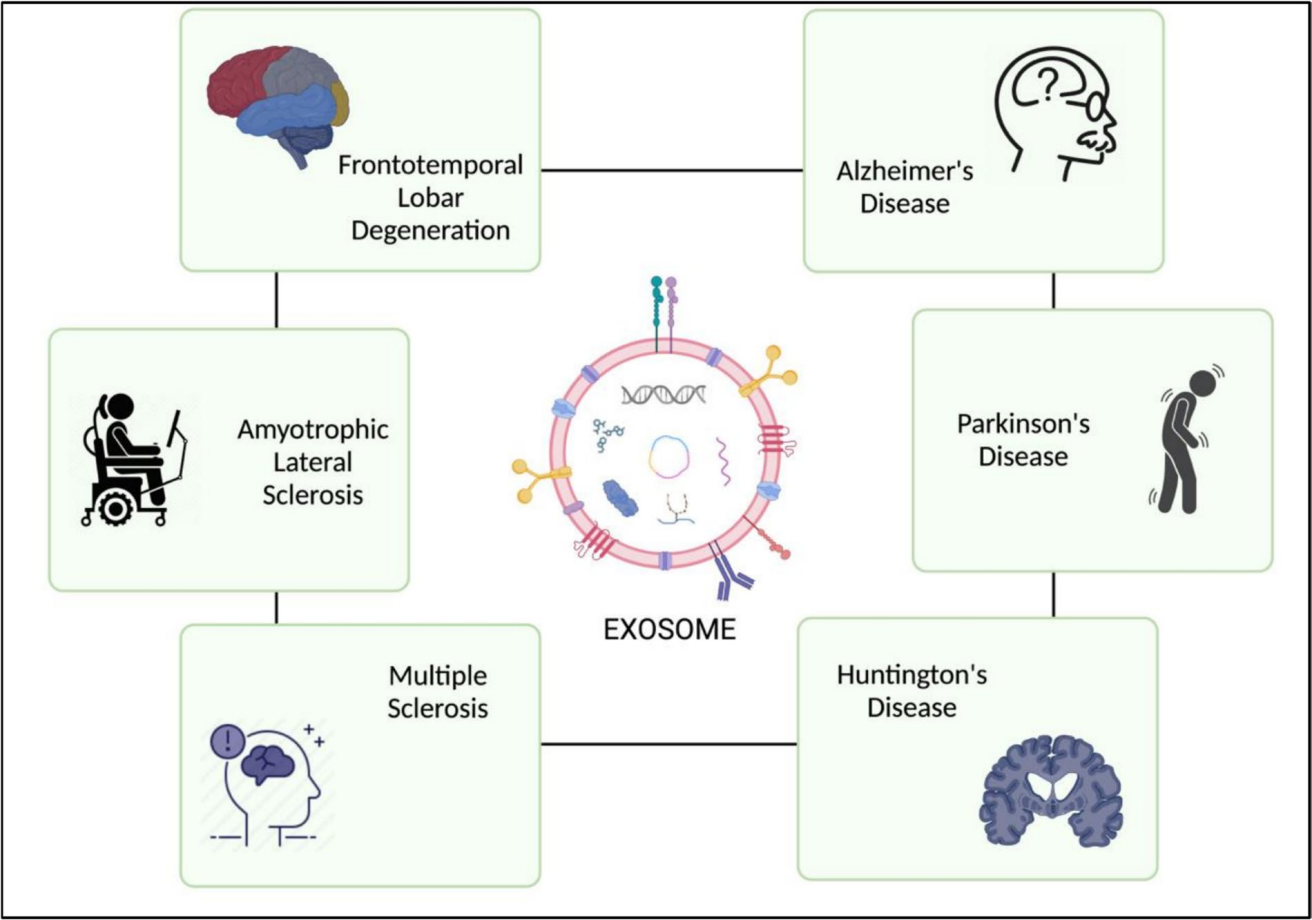
Schematic depiction of exosomes and their role in prominent neurodegenerative disorders, including Alzheimer’s disease, Parkinson’s disease, Huntington’s disease, amyotrophic lateral sclerosis (ALS), frontotemporal lobar degeneration (FTLD), and multiple sclerosis (MS). Exosomes are essential for intercellular communication as they transport macromolecules, including proteins, RNA, and lipids, positioning them as possible diagnostic indicators and therapeutic instruments in certain circumstances

Exosomes have been recognized for their potential as therapeutic carriers in neurodegenerative diseases due to their unique ability to transport bioactive compounds across cellular barriers and modulate various cellular processes [131, 132]. Their adaptability as therapeutic agents can be classified into two main categories: the inherent effects resulting from their natural molecular composition, and the targeted therapeutic cargo that can be incorporated into them [19]. Through these mechanisms, they not only offer direct neuroprotective and anti-inflammatory effects but also function as customizable delivery vehicles for targeted molecular treatments. Several research in the literature have demonstrated that exosome-based treatments had direct and significant effect on functional recovery such as behavioral, physical, and cognitive improvements in in vivo models and clinical trials [133–135]. Specific literature examples of therapeutic usage and consequences of exosome-based therapies are illustrated in Fig. 3 and Table 2.

**Table 2:** Therapeutic effects and outcomes of exosomes in neurodegenerative diseases

Types of NDS	Exosome source	Therapeutic molecule	Mechanism of action	Therapeutic outcome	Reference
Parkinson’s disease	Human umbilical cord mesenchymal stem cell	Brain-derived neurotrophic factor (BDNF)	Suppression of apoptosis and ferroptosis; promotion of neuronal cytoskeletal stability	Facilitates neuronal repair and regeneration by augmenting nigrostriatal pathway functionality and has neuroprotective impact	[145]
Parkinson’s disease	Dendritic cells	Rabies virus glycoprotein (RVG)–modified exosomes laden with shRNA-minicircles	Downregulation of alpha-synuclein expression	Decrease in alpha-synuclein aggregations, reduction in loss of dopaminergic neurons in substantia nigra pars compacta	[146]
Parkinson’s disease	Human umbilical cord mesenchymal stem cell	miR-133b	Decreases the phosphorylation level of Tau protein by targeting RhoA-ROCK signaling pathway	Improvement in motor function, reduced depression, enhanced dopaminergic neuronal function	[147]
Alzheimer’s disease	Adipose-derived stem cell	Circ-Epc-1	Decrease in M1 microglial differentiation, promotion of M2 microglial differentiation	Decrease in inflammatory cytokine expressions and decrease of apoptosis in hippocampal neurons, improving cognitive function by neuronal damage decrease	[136]
Alzheimer’s disease	Macrophage	Mannose-modified exosomes laden with gemfibrozil	Decrease in amyloid fibril formation, promotion of Aß intake by microglia, acceleration of lysosome-mediated Aß clearance	Mitigation of cognitive impairment and alleviation of neuronal damage	[148]
Alzheimer’s disease	Rat-bone marrow mesenchymal stem cells and human embryonic kidney 293 cells	miR-29b	Downregulation of BACE1, enhancement of PKA/CREB signaling inhibition of neuronal apoptosis	Improvement in spatial learning and memory, reduction of Aβ-induced cognitive deficits	[149]
Multiple sclerosis	Mesenchymal stem cell	LJM-3064 aptamer	Immunomodulation	Reduction in demyelination region—brain damage—and inflammatory cell infiltration into central nervous system	[150]
Multiple sclerosis	Macrophage	Resveratrol	Inhibition of NF-κB pathway	Decrease in inflammatory responses, inflammatory cell infiltration in central nervous system, demyelination	[144]
Huntington’s disease	Human embryonic kidney cells	miR-124	Binding to RE1-silencing transcription (REST) mRNA	Reduction in REST expressions in striatum; no production of significant behavioral improvement	[143]
Huntington’s disease	Neural stem cells	DNAJB6	DNAJB6 binds to misfolded polyglutamine (polyQ) and facilitates their transfer to other chaperones	Decrease in polyQ aggregation in stratium	[151]
Spinocerebellar ataxias	Human olfactory ensheathing cells	has-miR-6780	Inhibition of PI3KR5-related autophagy inhibition	Decrease in expression of polyQ, increase in expression of LC3II/I and improved motor coordination, getting benefit from butylidenephthalide	[152]
Spinocerebellar ataxias	HEK293 cells	miR-25 and miR-181a in RVG-modified exosomes	Inhibition of mutATXN3 expression	Decrease in mutATXN3 mRNA and improvement in motor ability and balance	[153]
Amyotrophic lateral sclerosis	Mesenchymal stem cells	miR-467 and miR-466q	Reduction in the activation of the p38 MAPK pathway	Beneficial anti-inflammatory effect on pro-inflammatory microglia	[154]

### Intrinsic Therapeutic Effects of Exosomes

Exosomes exhibit inherent therapeutic potential due to their unique chemical composition and the bioactive compounds they intrinsically generate from their progenitor cells. This naturally occurring composition consists of lipids, surface proteins, and endogenous RNAs, all of which work together to make it possible for exosomes to participate in the control of cell signaling and the regulation of immunological function within the nervous system. In the context of neurodegenerative diseases, where chronic inflammation, oxidative stress, and disrupted signaling contribute to the progressive loss of neuronal function, these intrinsic effects are particularly beneficial. For example, Circ-Epc1, a circular RNA that affects microglial polarization, is naturally present in exosomes generated from adipose-derived stem cells (ASCs) [136]. In neurodegenerative conditions such as Alzheimer’s disease, where neuroinflammation intensifies neuronal injury, Circ-Epc1 has been demonstrated to facilitate a transition from the pro-inflammatory M1 microglial phenotype to the anti-inflammatory M2 phenotype. This transition results in a decrease in the production of pro-inflammatory cytokines and a decrease in apoptosis within hippocampal neurons, thereby promoting neuroprotection and possibly reducing the progression of the disease. Furthermore, exosomes from macrophages have been shown to have an inherent capacity to improve the clearance of amyloid-beta (Aβ) in Alzheimer’s models [137]. They promote Aβ uptake and lysosomal damage through natural interaction with microglia, thereby reducing the harmful accumulation of amyloid plaques, a characteristic of Alzheimer’s pathogenesis. This natural clearance mechanism emphasizes the inherent anti-amyloidogenic characteristics of exosomes and highlights their potential as therapeutic agents for treating critical clinical aspects of neurodegenerative disorders. Besides their impact on neuroinflammation and amyloid-beta clearance, they intrinsically facilitate cell-to-cell communication, enabling the modulation of neuronal survival pathways [138]. Their lipid bilayer composition and related proteins enable interactions with cellular receptors in target cells, triggering signaling cascades that enhance cellular resilience to oxidative stress and other neurotoxic threats. These intrinsic functions render exosomes both adaptable and inherently suited to the treatment requirements of neurodegenerative disorders. The range of intrinsic therapeutic effects documented in recent research is summarized in Table 2.

### Therapeutic Impact of Exosome Cargo Molecules

Exosomes can transport and deliver unique therapeutic cargo molecules that can be externally loaded to target specific disease pathways, greatly increasing their therapeutic potential [139, 140]. The cargo molecules comprise various RNA types (including microRNAs and messenger RNAs), proteins, and small molecules that can be selected for their ability to modulate disease-related processes, such as apoptosis, inflammation, and protein aggregation [141]. By means of specific loading of these compounds, exosomes function as precise delivery systems that enhance their therapeutic potential beyond their inherent characteristics [142].

For instance, miR-124 is loaded into exosomes and used in models of Huntington’s disease [143]. miR-124 has the ability to downregulate RE1-silencing transcription (REST) mRNA, a regulator linked to Huntington disease. By diminishing REST levels, exosomes containing miR-124 alleviate detrimental gene expression alterations linked to the disease, providing a gene-targeted strategy that addresses the fundamental causes of neuronal toxicity in Huntington’s. On the other hand, exosomes loaded with anti-inflammatory agents exhibit considerable therapeutic advantages, especially in neuroinflammatory conditions such as multiple sclerosis. Macrophage-derived exosomes containing resveratrol suppress the NF-κB signaling pathway in multiple sclerosis models, resulting in decreased inflammatory cytokine production and mitigated demyelination in the central nervous system [144]. The attenuation of neuroinflammation is essential for decelerating the advancement of MS, wherein immune cell infiltration and inflammation significantly influence disease severity. In addition to RNA and small molecule delivery, exosomes can be modified to transport neurotrophic substances that promote neuronal survival and repair processes. For instance, exosomes containing brain-derived neurotrophic factor (BDNF) generated from human umbilical cord mesenchymal stem cells (MSC) have demonstrated potential in models of Parkinson’s disease [145]. BDNF facilitates neuronal regeneration, diminishes apoptosis, and safeguards neurons against ferroptosis, a form of cell death linked to iron accumulation and oxidative stress. Exosomes stimulate the regeneration of dopaminergic neurons and improve motor function recovery in Parkinson’s disease animals by delivering BDNF directly to the damaged regions. In conclusion, the versatility and targeted therapeutic capabilities of exosome cargo molecules—which encompass specific miRNAs, proteins, and other therapeutic agents—are critically dependent on their impact. These cargo molecules allow exosomes to have a highly specific impact on gene expression and cellular pathways within recipient cells, providing a precise method of addressing the mechanisms of neurodegenerative diseases. Tablo 2 provides a comprehensive overview of the therapeutic cargo molecules and their documented effects in recent studies.

## Future Trends and Potential of Exosomes as Therapeutic Agents

Exosomes present significant potential as therapeutic agents in neurodegenerative diseases, providing unique opportunities for drug delivery, overcoming biological barriers such as the blood–brain barrier (BBB), and contributing to clinical applications. This section discusses emerging trends and prospective breakthroughs.

### Exosomes as Targeted Drug Delivery Vehicles

Exosomes exhibit inherent characteristics like biocompatibility, stability, low toxicity, and reduced immunogenicity, rendering them optimal candidates for drug administration. These vesicles inherently transport various cargo, such as DNA, RNA, proteins, lipids, and metabolites, allowing them to influence biological processes in destination cells [155]. Moreover, exosomes can be modified to transport therapeutic agents with greater precision, hence augmenting their clinical efficacy [156, 157]. Recent developments have shown that therapeutic miRNAs, like miR-124 and miR-520f-3p, can be successfully loaded into exosomes to treat 6-hydroxydopamine (6-OHDA)-induced Huntington’s disease in mice, improving neuronal survival and reducing cell death [158]. The surface of exosomes is enriched with proteins, including transmembrane proteins, integrins, CD63, CD9, and CD81, which can function as both biomarkers and targeting ligands [159]. In addition to natural targeting, scientists have effectively modified exosomal surfaces chemically, physically, and genetically [160]. The lipid bilayer can be coupled with peptides, ligands, antibodies, chimeric antigen receptors (CARs), and DNA/RNA aptamers to improve the functionality and selectivity of exosomes [161]. For instance, in an in vivo model of Alzheimer’s disease, exosomes containing RVG peptides and a genetically engineered CD10 variant demonstrated enhanced efficacy in targeting amyloid-beta aggregates and improved hippocampal delivery. Additionally, the CD10 variation increased the production of anti-inflammatory cytokines, providing a potentially effective therapy strategy for Alzheimer’s disease [162]. On the other hand, utilizing cutting-edge imaging methods as photoacoustic imaging, fluorescence imaging, bioluminescence imaging, radionuclide imaging, CT scans, and magnetic resonance imaging allows for the targeted delivery and in vivo monitoring of exosomes [163]. For example, scientists created an imaging mouse model to detect the long-term accumulation of exosomes in vivo using bioluminescence resonance energy transfer (BRET) technology. Cancer cells expressing CD63-Antares2, which emits luminescence, were implanted into mice to provide a xenograft animal model in which cancer-targeting medications can be investigated as an exosome imaging tool [164]. In another study, researchers created a sensor utilizing fluorescence resonance energy transfer (FRET) technology to track conformational changes. They focused on exosomal EGFRs, and their sensor “ExoSen (exosome sensor)” facilitated the quantification of exosomal EGFRs in both in vivo and in vitro cellular investigations [165]. In another study, researchers employed 64Cu and 68 Ga with fluorescence dyes to observe exosome distribution in vivo and ex vivo utilizing PET and optical imaging [166]. These technologies provide precise tracking of exosome biodistribution, facilitating the enhancement of their therapeutic uses.

### Exosomes as Key Players in Crossing the Blood–Brain Barrier

The BBB is a selective barrier comprised of various cell types, including endothelial cells, pericytes, astrocytes, and microglia. This barrier comprises tight junctions (TJs) and adherens junctions (AJs) between cells, safeguarding the central nervous system (CNS) from macromolecules (e.g., polypeptides, nucleic acids, and proteins), while over 98% of small molecules and immune cells originate from blood circulation [167, 168]. Despite neurological diseases being the second leading cause of death globally, the systemic administration of diagnostic and therapeutic agents has been impeded in achieving efficacy due to the presence of the BBB [169].

Exosomes penetrate cells via paracellular and transcellular routes while passing through the BBB. In paracellular routes, passive diffusion serves as the primary mechanism for exosome translocation. Since this form of crossing is actualized in TJs and many exosomes are higher than these openings, further research is needed; nonetheless production of inflammatory cytokines and certain physiological situations can cause this situation [170]. Normal exosomal transport into brain tissues occurs via direct fusion, macropinocytosis, clathrin-mediated endocytosis, caveolin-mediated endocytosis, and phagocytosis [171]. For instance, in a co-culture setup that resembled the blood–brain barrier, ginseng-derived exosomes demonstrated effective penetration across closely spaced epithelial cells, such as C6 glioma cells and brain capillary endothelial cells (BCECs). The research indicated that ginseng-derived exosome-like nanoparticles (GENs) may serve as prospective agents to impede glioma progression and regulate tumor-associated macrophages (TAMs), demonstrating anti-tumor immune responses and inhibition of tumor progression [172]. After entrance of exosomes into cells, they are guided to endosomes or lysosomal destruction and lysosomal degradation of exosomes regulates downstream processes [173].

Furthermore, it has been demonstrated that the integrity of the blood–brain barrier and the physiological condition of the brain are related to exosomal blood–brain penetration [174]. In a study, serum-derived exosomes from healthy rats were given to a brain microvascular endothelial cell type (bEnd.3) that was lacking oxygen and glucose and to rats that had a middle cerebral artery occlusion (MCAO). Serum exosomes were discovered to inhibit LC3B-mediated autophagy and reduce apoptosis in cerebral ischemic/reperfusion rat striatum by boosting the Bcl-2/Bax ratio and decreasing the expression of cleaved caspase-3 in the cortex. By preventing endothelial cell death and autophagy-mediated tight junction protein degradation, this study demonstrated that serum exosomes preserve the integrity of the blood–brain barrier [175]. In a separate study, researchers assessed quiet (Q-Neu) and activated neutrophil (A-Neu) exosomes in a transient middle cerebral artery occlusion-hemorrhagic transformation (tMCAO-HT) model, measuring permeability and trans-endothelial electrical resistance (TEER) to evaluate blood–brain barrier (BBB) integrity. The miRNA profiles of both neutrophils indicated that genes associated with the positive regulation of endothelial barrier formation, cell junctions, interactions between the extracellular matrix and receptors, and the VEGF pathway were targeting. Their findings indicated that Q-Neu exosomes do not significantly affect BBB integrity; nevertheless, the exosomal trafficking of miRNAs from A-Neu-derived exosomes may exacerbate BBB injury [176]. On the other hand, a recent study demonstrated that exosomes obtained from human induced pluripotent stem cell–derived neural stem cells (hiPSC–NSC–Exo) enhance blood–brain barrier function and reduce leukocyte infiltration after intracerebral hemorrhage. The intra-nasal administration of hiPSC–NSC–Exo led to a reduction in monocyte chemoattractant protein-1 production, mediated by the activation of the PI3K/AKT pathway in astrocytes [177]. Overall, the important ability of exosomes to traverse the blood–brain barrier and their essential functions in preserving their integrity will expand opportunities in the field of neurodegenerative disease research, accompanied by the emergence of novel methodologies and approaches.

### Exosome-Based Therapies in Clinical and Pre-clinical Trials

The majority of exosome-based clinical research on neurodegenerative illnesses is conducted on stem cells because of their immunosuppressive and regenerative capabilities, which have shown promise in recent years [178]. According to a recently published study, miR-29 expression is considerably reduced in Alzheimer’s disease (AD) patients, indicating that it plays in disease pathogenesis. To investigate therapeutic possibilities, researchers introduced miR-29 into target cells and collected exosomes produced by those cells. Rat bone marrow mesenchymal stem cells and HEK-293 T cells were transfected with recombinant expression vectors that carried miR-29a or miR-29b precursor sequences. The modified exosomes were examined for their capacity to ameliorate the pathogenic consequences of amyloid-β (Aβ) peptide in a rat model of Alzheimer’s disease. It was revealed that injecting miR-29-containing exosomes into the CA1 region of the rat brain reduced Aβ-related memory and spatial learning impairments [149]. In another study, exosomes generated from mesenchymal stem cells (MSC-exosomes) were co-cultured with human neural cells from familial Alzheimer’s disease and subsequently injected into transgenic mice. Behavioral assessments performed on these mice demonstrated notable enhancements in memory, cognitive abilities, and cerebral glucose metabolism. In cell culture assays, MSC-derived exosomes decreased Aβ expression and restored genes associated with memory and plasticity. It was determined that MSC-exosomes provide a promising cell-free therapeutic alternative for the treatment of Alzheimer’s disease [179]. Another recent study investigated the potential therapeutic benefits of adipose-derived stem cell extracellular vesicles (ASC-EVs) for patients with spinal muscular atrophy (SMA). SMNΔ7 animals, a severe SMA type II model, were given intracerebroventricular injections of isolated murine ASC-EVs during the neonatal phase. When compared to controls, the treatment group had better motor function and less apoptotic activation, according to behavioral tests and histological investigations. ASC-EVs were found to effectively replicate the neuroprotective effects of their progenitor mesenchymal stem cells, making them potential candidates for SMA therapy, either alone or in combination with drugs [180]. In another study, human adipose–derived stem cell–derived exosomes (ADSC-exo) were investigated as therapeutic agents in the realm of amyotrophic lateral sclerosis. ADSC-exo was administered to primary neuronal stem cells isolated from wild-type and G93A ALS mice during neuronal differentiation by researchers. The treated cells exhibited a substantial decrease in mutant SOD1 aggregation in the cytoplasm compared to the untreated ALS control cells by the tenth day of differentiation. Furthermore, the phospho-CREB/CREB ratio and PGC-1α expression levels normalized as a result of ADSC-exo treatment, indicating that exosomes may be able to protect cells from the aberrant mitochondrial protein expression that is associated with ALS [181]. Similarly, another study found that exosomes from murin adipose–derived stromal cells can treat NSC-34 cells that are like motoneuron and were transfected temporarily or permanently with human mutant SOD1 genes (G93A, G37R, or A4V). Cells maintained under oxidative stress conditions induced by H₂O₂ showed markedly enhanced viability suggesting the protective effect against to oxidative damage, a critical factor in ALS pathogenesis, when treated with exosome therapy [182].

Apart from the pre-clinical studies, clinical trials utilizing exosomes in NDDs have also being conducted. In a study, researchers delivered mesenchymal stem cell–secreted exosomes (MSCs-Exos) intravenously to Alzheimer’s disease (AD) patients, which previously eased the condition in mice models. This is the first clinical trial in patients, with dose selection prior to phase II and phase III clinical trials. Researchers carried out this study to evaluate the safety and efficacy of allogenic human adipose MSCs-Exos in individuals with mild to moderate Alzheimer’s disease (AD). The amount of amyloid or tau that was deposited did not change significantly. However, the volume of the hippocampus shrunk in people who received a medium dose of exosomes. The study established a suitable dosage for the internasal administration of MSCs-Exos, which is safe and well-tolerated [183]. In another phase I clinical trial, researchers administered allogenic exosomes derived from human umbilical cord mesenchymal stem cells (HUC-MSCs) intrathecally to patients with subacute spinal cord damage (SCI) to assess safety and possible efficacy. Neurological assessments were conducted prior to the application, and the intrathecal delivery of allogeneic HUC-MSC exosomes was demonstrated to be safe. Furthermore, the study indicated possible clinical and functional enhancements in patients with subacute spinal cord injury [178].

In conclusion, exosome-based therapeutics have shown promise in clinical trials, and the recent studies are given in Table 3. However, additional research and advanced clinical studies (phase II/III) are required to confirm their efficacy and safety. This next generation therapeutics may serve as a great potential to successfully address the growing incidence of neurodegenerative illnesses.

**Table 3:** Clinical trials of exosomes used in neurodegenerative disease

Clinical trial ID or Pubmed ID	Dates	Type of disease	Therapeutic agent/diagnostic biomarker	Source of exosome	Status	Sponsor
NCT06598202	S: 2024.12.01 C: 2026.05.30	Amyotrophic lateral sclerosis	Exosomes derived from human umbilical cord blood mesenchymal stem cells	Human umbilical cord blood derived mesenchymal stem cells	Recruiting phase I/II	Xuanwu Hospital, Beijing
NCT04388982	S: 2020.07.01 C: 2022.08	Alzheimer’s disease	Exosomes derived from allogenic adipose MSCs	Allogenic adipose MSCs	Unknown phase I/II	Ruijin Hospital
NCT06501469	S: 2022.03.23 C: 2030.03.25	Parkinson’s disease	Not stated	Blood	Recruiting	Anonym
PMC7963893	2021	Alzheimer’s disease	miR-193b	Serum	Completed	National Natural Science Foundation of China
PMC5888398	2018	Alzheimer’s disease	Specialized synaptic proteins	Neurons	Completed	Michael J. Fox Foundation for Parkinson’s Research, the Alzheimer’s Association, Alzheimer’s Research United Kingdom, and the Weston Brain Institute
PMC6459135	2019	Parkinson’s disease	Brain insulin-signaling proteins	Neurons	Completed	University College London

## Discussion

Exosomes have garnered significant attention as both biomarkers and therapeutic tools in the field of neurodegenerative diseases, offering unique, non-invasive approaches for diagnosis, monitoring, and treatment. Unlike traditional biomarkers, which can be invasive or costly, exosome-based strategies promise accessible and personalized insights into disease mechanisms [184, 185]. This review evaluated recent studies on exosome applications, highlighting their distinct properties and potential to reshape neurodegenerative disease research while underscoring the challenges that must be addressed for clinical translation.

One critical aspect across studies is the unique protein and nucleic acid cargo contained within exosomes isolated from neurodegenerative disease patients [186]. These exosomes, derived from various body fluids—including blood, cerebrospinal fluid, urine, saliva, sweat, and tears—carry disease-specific markers that reflect the pathological state of the brain, offering a valuable foundation for biomarker development [187]. Exosomal biomarkers, particularly those found in accessible fluids like blood, saliva, sweat, and tears, stand out as non-invasive, cost-effective, and rapid alternatives to traditional detection methods like brain imaging or cerebrospinal fluid punctures, which can be invasive, costly, and time-consuming [188]. Each body fluid, however, presents unique logistical and technical considerations [189]. Blood offers a high yield but requires careful purification due to its rich protein content, while cerebrospinal fluid provides the most direct link to central nervous system activity but is invasive to collect. Saliva, sweat, and tears represent promising non-invasive options, with saliva offering easy and routine collection and wearable devices potentially enabling sweat sampling, though all may yield lower exosome concentrations [190]. For example, specific proteins associated with Alzheimer’s disease have been detected in circulating exosomes from both blood and cerebrospinal fluid, demonstrating their potential as accessible tools for real-time disease monitoring [191]. Moreover, this disease-specific profiling offers promising avenues for early diagnosis and tracking disease progression, which is critical in neurodegenerative conditions. However, methodological variations—such as differing isolation techniques across body fluids and assay sensitivities—can impact biomarker accuracy and reliability [192]. Studies have shown that while some isolation methods maximize exosome yield from fluids like blood or cerebrospinal fluid, they may compromise purity, raising concerns about biomarker specificity [193]. To enhance reproducibility and clinical applicability, future research should prioritize standardizing protocols for exosome isolation and analysis across different body fluids [194].

On the other hand, despite attempts to enhance exosome extraction from diverse biofluids, considerable obstacles persist in standardizing methods to guarantee reproducibility and clinical relevance. For example, in a clinical trial, plasma BACE1-AS levels showed no significant difference between AD patients and the healthy group [195]. However, in a prior research, the situation was exactly the opposite, showing a great difference between healthy control group and AD patients [196]. Yield variation is a significant issue, as certain biofluids intrinsically yield lower exosome concentrations, requiring highly sensitive testing techniques. Furthermore, variations in protein content among biofluids can hinder isolation efficiency, necessitating additional purification processes for high-protein fluids such as blood to preserve biomarker specificity. Specifically, one issue arises from the complex origin of CSF and peripheral blood exosomes. In a clinical trial searching if miR-193b detection in exosomes is a potential diagnostic biomarker in AD, the variability in the source of exosomes can lead to ineffective isolation and detection of high-miR-193b exosomes, resulting in false negative results in patients [197]. Hence, future research must concentrate on creating standardized, high-yield, and reproducible isolation methodologies for exosomes, specifically designed for each biofluid source, to maximize their utility as diagnostic tools.

Advancements supporting the use of exosomes as biomarkers are closely tied to the strengths and limitations of various detection technologies. Microfluidic platforms, which enable rapid and precise exosome isolation, are particularly advantageous due to their low cost and suitability for small sample volumes; however, the lack of full standardization may affect result consistency across studies [198]. Nanoparticle tracking analysis provides highly accurate measurements of exosome size and concentration, yet its high cost and specialized technical requirements limit broader use [199]. Fluorescence and optical microscopy techniques allow visualization of specific surface proteins, offering an advantage in multi-marker detection, though these methods may incur additional costs and time due to the need for specific staining [200]. Meanwhile, some highly sensitive technologies struggle to completely differentiate exosomes from other cellular particles, potentially impacting biomarker purity, which is critical for clinical diagnosis. Considering the heterogeneity of exosomes, advanced detection methods that distinguish exosome subtypes by cellular origin could enhance biomarker precision, particularly for neurodegenerative conditions where neural-derived exosomes hold significant diagnostic value [201]. Developing low-cost, high-precision, and portable solutions in exosome detection technologies will be essential for making exosomes more accessible in clinical diagnostic and monitoring settings [202]. Additionally, integrating these detection techniques with multi-omics approaches, such as proteomics and transcriptomics, could deepen our understanding of exosomal content and improve disease-specific profiling, potentially making lab-on-a-chip devices suitable for point-of-care diagnostics and routine disease monitoring across various body fluids [203]. Portable platforms, such as lab-on-a-chip devices, represent a promising future in exosome detection across various body fluids [204, 205].

In addition to their role as biomarkers, exosomes have shown considerable promise as delivery vehicles for therapeutic agents, particularly for their ability to cross the blood–brain barrier (BBB) with ease. This natural capacity distinguishes exosomes from other nanoparticle-based delivery systems, which often encounter significant challenges in penetrating the BBB and reaching target neural tissues [206]. Leveraging this advantage, recent studies have encapsulated therapeutic molecules, including siRNA, small-molecule drugs, and neuroprotective agents, within exosomes isolated from body fluids like blood and cerebrospinal fluid to deliver targeted treatments to neurodegenerative pathways [207]. Compared to conventional delivery approaches, exosomes offer a minimally invasive, biocompatible, and potentially cost-effective strategy for targeted therapy, making them highly feasible for clinical practice [208]. Advances in exosome engineering have further expanded their versatility, enabling the delivery of complex therapeutic cargos such as CRISPR/Cas9 for gene editing or anti-inflammatory agents aimed at modulating neuroinflammation [209, 210]. Despite these promising developments, challenges remain regarding targeting specificity and off-target effects; ensuring that exosomes can selectively deliver therapeutic agents to diseased cells without affecting healthy tissue is critical [211–213]. Long-term biocompatibility and stability are also essential considerations, given the delicate nature of neural tissue and the need for consistent therapeutic efficacy [214, 215]. Addressing these issues through rigorous preclinical and clinical studies will be vital to advancing exosome-based delivery systems toward safe and effective use in treating neurodegenerative diseases.

Furthermore, differentiating exosome profiles from various neurodegenerative diseases, such as Alzheimer’s, Parkinson’s, and ALS, is essential for both diagnostic and therapeutic applications. Distinct exosome profiles have been observed in patients with each disease across different body fluids, offering a valuable method for differential diagnosis [216]. For example, variations in exosomal content between CSF and blood samples have been linked to specific pathologies, suggesting that fluid-specific markers could significantly enhance diagnostic accuracy [124]. This specificity underscores the potential of exosome-based diagnostics to provide more personalized, precise, and minimally invasive tools compared to traditional biomarkers. Differentiating between these diseases is crucial, as they often present with overlapping symptoms yet require distinct treatment approaches. However, while some studies demonstrate high sensitivity and specificity of exosomal biomarkers, variability in exosome content across patients, disease stages, and body fluids presents a challenge for achieving consistent diagnostic accuracy [217]. Addressing this heterogeneity will require technological advancements and standardized protocols, especially in cross-study comparisons. More comprehensive studies with larger sample sizes and diverse cohorts are needed to clarify these variations and strengthen the diagnostic validity of exosomes, ultimately advancing their application in personalized neurodegenerative disease management [218].

As exosome-based diagnostics and therapies advance rapidly towards clinical application, their complex nature and potential risks underscore the necessity for rigorous regulatory oversight in a largely undeveloped regulatory landscape, posing significant challenges. Exosomes are not classified as advanced therapy medicinal products (ATMPs) unless used for gene therapy, complicating their regulatory pathways in Europe and the USA [219]. This classification ambiguity impacts regulatory strategies and development plans, emphasizing the need for clear guidelines for their use as biological medicinal products. The absence of standardized guidelines for exosome characterization, production reproducibility, and safety assessments further complicates the pathway toward clinical implementation [220]. Ethical considerations—particularly around patient consent and the long-term monitoring of exosome-derived treatments from body fluids—present additional challenges. The collection and analysis of body fluids must respect patient autonomy and privacy [221]. Moreover, long-term monitoring is necessary to assess the safety and efficacy of exosome-based treatments, raising ethical concerns about patient follow-up and data management [222]. Establishing robust regulatory frameworks that ensure safety, efficacy, and ethical standards will be critical in advancing exosome-based applications from research to practice. Achieving this requires a high level of cooperation among policymakers, researchers, clinicians, and regulatory authorities to develop comprehensive standards that address these regulatory and ethical concerns while fostering innovation in a manner that prioritizes patient safety and ethical integrity [220]. Balancing innovation with patient safety and ethical standards is crucial for the successful clinical implementation of these therapies.

Finally, as regulatory and ethical frameworks take shape, the production of exosomes for therapeutic purposes continues to face significant technical challenges, particularly in ensuring yield, purity, and consistency across various body fluids [223]. Differences in isolation methods—whether from blood, cerebrospinal fluid, urine, or saliva—contribute to variability in exosome quality, which in turn impacts their therapeutic efficacy [224]. Techniques such as ultracentrifugation, filtration, and immunoaffinity capture each have distinct advantages and limitations based on the fluid source, yet this variability underscores the need for a standardized, reproducible approach to exosome isolation [225]. Establishing such standardization would reduce costs, streamline procedures, and improve consistency, ultimately making exosome-based therapies more accessible and clinically viable [226]. Reaching a consensus on the most effective techniques to isolate high-quality exosomes with therapeutic potential from each fluid type is essential. Standardized protocols and rigorous testing will be crucial for optimizing exosome production to meet the demands of clinical application and ensure reliable patient outcomes.

In conclusion, exosomes derived from various body fluids present a transformative opportunity in the diagnosis and treatment of neurodegenerative diseases. Their unique ability to serve as non-invasive, accessible, and rapid biomarkers, coupled with their potential as targeted therapeutic delivery vehicles, positions exosomes as a critical tool in advancing personalized medicine. However, to realize this potential fully, challenges surrounding standardization, targeting specificity, regulatory pathways, and ethical considerations must be systematically addressed. Through collaborative research efforts, technological innovation, and the development of robust regulatory frameworks, exosome research can transition from bench to bedside, paving the way for more effective and accessible care for neurodegenerative disease patients. Continued investment in this area holds the promise of transforming patient outcomes and shaping the future of neurodegenerative disease management.

## Current Challenges and Future Perspectives

Despite the promising potential of exosomes as both biomarkers and therapeutic agents in neurodegenerative diseases, significant challenges remain, including the standardization of isolation techniques, exosome heterogeneity, risk of immune activation, challenges in large-scale production for clinical applications, short circulation half-life, long-term safety, and the need for standardized regulatory frameworks for exosome-based therapeutics.

Firstly, the lack of standardized protocols for isolating and characterizing exosomes poses a fundamental challenge [227]. Current methods, such as ultracentrifugation and immunoaffinity capture, vary widely in terms of purity, yield, and exosomal integrity, leading to inconsistencies across studies and limiting reproducibility [228]. Secondly, exosome heterogeneity further complicates the therapeutic landscape [229]. Exosomes originating from different cell types exhibit varied molecular profiles, which hinders the identification of disease-specific biomarkers crucial for early diagnosis and targeted therapy [230]. Moreover, immune activation remains a significant concern, particularly in the context of allogeneic or engineered exosomes [231]. Although exosomes generally have a lower immunogenicity than synthetic nanoparticles, research has indicated that specific exosomal components, including RNA cargo and surface proteins, can elicit immune responses, potentially restricting their long-term therapeutic applications [178]. Additional research is required to reduce immunogenicity while maintaining therapeutic efficacy. Furthermore, scaling up exosome production for clinical applications appears to be a substantial obstacle. Strict control over batch-to-batch consistency, purity, and functional integrity is necessary for large-scale production, but current isolation techniques are challenging to implement [232]. The development of high-throughput, standardized manufacturing processes is necessary to facilitate clinical translation, as existing methods, such as ultracentrifugation and size-exclusion chromatography, are not readily scalable [233]. The short half-life of exosome-based therapies in circulation is another critical limitation that considerably affects their efficacy in delivering therapeutic cargo to target sites [234]. Studies have demonstrated that EVs derived from human platelets have a circulation half-life of approximately 5.5 h [235], whereas systemically administered exosomes exist in the bloodstream for only about 2 h before being rapidly cleared [236]. Another issue diminishing the therapeutic efficacy of exosomes is their rapid clearance, which restricts the adequate duration of their interaction with their target tissues [237]. To overcome this limitation, strategies such as encapsulation within biomaterial-based carriers or surface modifications, have been proposed to increase the therapeutic potential of exosomes and extend their circulation time [238]. In this context, the combination of the liposomal formulations may provide benefit from established stability and controlled release properties, while engineered exosomes offer superior biocompatibility and endogenous targeting capabilities [239]. When it comes to the long-term safety, the stability of exosomes and their potential adverse effects address further evaluation [240]. Despite the fact that patient-specific exosomes improve biocompatibility, their production is still resource-intensive and scaling up remains challenging [241]. Additionally, it is necessary to establish standardized regulatory frameworks, as the current guidelines may not adequately address the distinctive characteristics of exosomes, necessitating modifications for clinical translation [242]. These above-mentioned interrelated issues highlight the necessity for ongoing innovation and collaboration within scientific, regulatory, and clinical domains to fully exploit the therapeutic promise of exosomes in NDDs.

On the other hand, prospects for exosome-based applications in NDDs are promising, driven by advances in microfluidic technologies, CRISPR/Cas system, next-generation sequencing (NGS), artificial intelligence (AI), bioengineering modifications, personalized exosome production, and the discovery of disease-specific biomarkers. Microfluidic technologies offer new pathways for efficient exosome isolation, allowing for high-purity separation directly from complex biological fluids and potentially paving the way for integrated diagnostic devices at point-of-care settings [243]. CRISPR/Cas system is guided by an engineered RNA which makes genetic improvements in faulty region of a DNA. For example, a research study reported successful epigenome editing using this method, demonstrating a reduction in BACE1 expression in an Alzheimer’s disease model [244]. NGS and quantitative PCR are accelerating the discovery of disease-specific exosomal biomarkers by enabling precise molecular profiling of exosome cargo, including RNA and protein content; this profiling is crucial for early diagnosis and personalized monitoring of disease progression [245]. AI and machine learning (ML) further enhance the field by providing powerful tools for analyzing exosomal data, uncovering correlations within molecular profiles that may indicate disease stages or predict therapeutic outcomes [246]. Furthermore, these AI algorithms have been used as biomarker detectives, analyzing proteome signature to identify cancer-related exosomes [247]. Additionally, bioengineering approaches, such as modifying exosome surface proteins or embedding specific ligands, are enhancing targeting specificity, allowing exosomes to deliver therapeutic molecules directly to affected neural cells, thereby minimizing off-target effects [12, 248]. Personalized exosome production also holds promise, as exosomes derived from patient-specific cells are more likely to achieve high biocompatibility and minimize immune response, supporting the growing interest in tailored therapeutic interventions [249]. Finally, with the discovery of disease-specific biomarkers, exosomes are expected to enable not only early and dynamic disease monitoring but also real-time evaluation of treatment efficacy, allowing clinicians to adjust as necessary to maximize therapeutic impact [250]. Collectively, these advancements indicate a future in which exosomes may facilitate a responsive and personalized precision medicine revolution, fundamentally transforming the diagnosis and treatment of neurodegenerative diseases.

## Conclusion

To close the gap between diagnosis and treatment, exosomes have become game-changing instruments in the study of neurodegenerative diseases. Their exceptional capabilities to transport biomarkers specific to diseases and to traverse the blood–brain barrier render them priceless for precise early detection and treatment. Despite the promising results of both preclinical and clinical investigations, there are still obstacles to overcome before these discoveries may be implemented in the clinic. These include issues with standardization, scalability, and regulatory frameworks. Applications based on exosomes have the potential to change the way neurodegenerative diseases are managed, leading to better patient outcomes and a move towards precision medicine, by encouraging collaboration across disciplines and making use of recent advances in molecular biology and bioengineering.

## Data Availability

No datasets were generated or analysed during the current study.

## Abbreviations

Aβ: Amyloid-beta
AD: Alzheimer’s disease
ALS: Amyotrophic lateral sclerosis
ApoBDs: Apoptotic bodies
ASC: Adipose-derived stem cell
AUC: Area under the curve
BBB: Blood-brain barrier
CNS: Central nervous system
CSF: Cerebrospinal fluid
EVs: Extracellular vesicles
FTLD: Frontotemporal lobar degeneration
HD: Huntington’s disease
HUC-MSCs: Human umbilical cord mesenchymal stem cells
MS: Multiple sclerosis
MSCs: Mesenchymal stem cells
MVB: Multivesicular body
NDDs: Neurodegenerative diseases
PD: Parkinson’s disease
RVG: Rabies virus glycoprotein
SCA: Spinocerebellar ataxia
SMA: Spinal muscular atrophy
